# Assessing stress levels in older adults during cognitive and motor testing to advance earlier dementia screening

**DOI:** 10.1093/geroni/igaf122.2516

**Published:** 2025-12-31

**Authors:** Bernardo Villa-Sánchez, Alexandra Reed, Brianna Branson, Elyana Chacon, Sydney Schaefer

**Affiliations:** Arizona State University, Tempe, Arizona, United States; Arizona State University, Tempe, Arizona, United States; Arizona State University, Tempe, Arizona, United States; Arizona State University, Tempe, Arizona, United States; Arizona State University, Tempe, Arizona, United States

## Abstract

Dementia is the second most feared diagnosis among adults, yet only a small percentage undergo cognitive testing for early signs during routine visits with primary care providers. It is possible that one reason for the low rate of routine screening may be patients’ resistance to testing, suggesting that anticipating or completing a cognitive assessment may induce stress in older adults. The primary purpose of this study was to determine the stress responses of older adults immediately prior to and during cognitive screening. This study also compared responses between a traditional (Montreal Cognitive Assessment, MoCA) and a novel (Brief Evaluation of Cognition and Daily Function, BEAN) assessment. Twenty-one unimpaired older adults were randomly assigned to either the MoCA or BEAN group and were initially blinded to their group assignment. Test-related stress was measured using subjective (STAI-6) and objective (salivary α-amylase and cortisol) methods at four time points: baseline, in anticipation of assessment type (immediately following unblinding), and then five and 30 minutes after test initiation. Contrary to our hypothesis, neither STAI-6 scores, α-amylase, nor cortisol values indicated test-related stress for either assessment relative to baseline, based on Bonferroni-adjusted repeated-measures ANOVAs. These results are important, as cognitive screening in both clinical care and clinical trials should have minimal stress responses. Future studies should explore test-related stress using continuous measures of stress response, such as electrodermal activity, particularly in patients with cognitive decline.

